# Association between depressive symptoms and poor sleep quality among Han and Manchu ethnicities in a large, rural, Chinese population

**DOI:** 10.1371/journal.pone.0226562

**Published:** 2019-12-19

**Authors:** Ru-Qing Liu, Michael S. Bloom, Qi-Zhen Wu, Zhi-Zhou He, Zhengmin Qian, Katherine A. Stamatakis, Echu Liu, Michael Vaughn, Wayne R. Lawrence, Mingan Yang, Tao Lu, Qian-Sheng Hu, Guang-Hui Dong

**Affiliations:** 1 Guangzhou Key Laboratory of Environmental Pollution and Health Risk Assessment, Department of Preventive Medicine, School of Public Health, Sun Yat-sen University, Guangzhou, Guangdong, China; 2 Department of Environmental Health Sciences, University at Albany, State University of New York, Rensselaer, New York, United States of America; 3 Department of Epidemiology and Biostatistics, School of Public Health, University at Albany, State University of New York, Rensselaer, New York, United States of America; 4 Department of Epidemiology, College for Public Health and Social Justice, Saint Louis University, Saint Louis, Missouri, United States of America; 5 Department of Health Management & Policy, College for Public Health and Social Justice, Saint Louis University, Saint Louis, Missouri, United States of America; 6 School of Social Work, College for Public Health and Social Justice, Saint Louis University, Saint Louis, Missouri, United States of America; 7 Department of Biostatistics and Epidemiology, Graduate School of Public Health, San Diego State University, San Diego, California, United States of America; 8 Department of Mathematics and Statistics, University of Nevada, Reno, Nevada, United States of America; KHANA, CAMBODIA

## Abstract

**Objectives:**

To estimate the relationship between sleep quality and depression, among Han and Manchu ethnicities, in a rural Chinese population.

**Methods:**

A sample of 8,888 adults was selected using a multistage cluster and random sampling method. Sleep quality was evaluated using the Pittsburgh Sleep Quality Index (PSQI). Depressive symptoms were assessed via the Center for Epidemiological Survey, Depression Scale (CES-D). Logistic regression was conducted to assess associations between sleep quality and depression.

**Results:**

The prevalence of poor sleep quality and depression in the Manchus (20.74% and 22.65%) was significantly lower than that in the Hans (29.57% and 26.25%), respectively. Depressive participants had higher odds ratios of global and all sub PSQI elements than non-depressive participants, both among the Hans and the Manchus. Additive interactions were identified between depressive symptoms and ethnicity with global and four sub-PSQI elements, including subjective sleep quality, sleep disturbance, use of sleep medication and daytime dysfunction.

**Conclusions:**

The findings revealed that the prevalence of poor sleep quality and depression among the Hans was greater than among the Manchus. Depression was associated with higher odds of poor sleep quality.

## Introduction

Worldwide, depression accounts for almost 9.6% of all total years lived with disability [1]. Additionally, depression is responsible for the highest burden of non-fatal disease worldwide, and is the fourth leading cause of overall disease overall [1, 2]. In Yu et al.’s recent population-based study of 18,994 Chinese adults in, the prevalence of depressive symptoms was as high as 16.3% [3]. Previous studies from Brazil and Canada reported links for depression with higher overall morbidity and premature mortality, as well as with reduced social interactions, less work productivity, and early retirement [4, 5].

Poor sleep quality is closely linked to depression [6, 7]. However, reported associations between depression and poor sleep quality have varied across study populations. Atalay et al. reported no significant correlations between patients’ insomnia scores and their depressive status [8]. Recently, a meta-analysis of thirty-four cohort studies, involving 172,077 participants, reported a statistically significant association between insomnia and an increased risk of depression [9].

Most previous studies of sleep quality and depression were conducted in western or developed countries, and focused on urban populations, while very few studies have been carried out in developing countries. In developing countries, citizens living in rural areas comprise a significant proportion of the population. In 2017, approximately 50.32% of people in China lived in rural agricultural regions [10], and limited data exist on sleep quality and depression in China’s rural population. Even population-based data on poor sleep quality are limited and inconsistent among Chinese adults, with prevalence reported as 11.9%-49.7% [11]. For example, Wang et al. recently reported that the sleep disorders were highly prevalent among elderly rural Chinese, with a 33.8% overall prevalence [12]. While Cao et al. recently reported a significantly higher prevalence of depressive symptoms among rural (34.0%) compared to urban (24.7%) Chinese [13]. Finally, a recent meta-analysis of 47 epidemiologic studies, showed that older Chinese adults in rural areas had a higher prevalence of sleep disturbances than their urban counterparts [14]. Overall, there is a need for more epidemiological studies in rural communities.

Additionally, there may be differences in associations between sleep quality and depression across ethnic populations. The Manchu minority is the third-largest ethnic group in China, numbering around 10.68 million and comprising approximately 0.8% of the total population [15]. Being descendants of nomadic herders, Manchus have a cultural background and lifestyle that often differs from the majority Han ethnic group, which may be associated with a greater risk for depression and poor sleep quality [16–19]. However, thus far, there are no data available to characterize the prevalence of depression and sleep disorders among Manchus in China.

The goals of this study were (1) to address the pending data gap by examining associations between sleep quality and depression in a rural Chinese population, and (2) to explore differences in the prevalence of depression and sleep quality among the Han and Manchu ethnic groups. These goals were a result of data exploration. These efforts may provide important guidance for clinicians to foster earlier recognition of depression and poor sleep quality among the rural Chinese population, to identify high risk subgroups, and for public health policy makers to design preventive strategies for minimizing the impact of depression and poor sleep quality in the rural Chinese population.

## Methods

### Study population

Participant recruitment was previously described in detail [20]. In brief, we conducted population-based cross-sectional surveys in the rural areas of Jinzhou, Liaoning province, northern China using a multi-stage, stratified clustering sampling protocol. Data were collected from May, 2012 to April, 2013.

Briefly, the sampling rural areas was stratified into five geographic zones: North, South, East, West and Central. Two townships were randomly selected from each of the South, East and West zones and one township was selected from each of the North and Central zones. Three or five villages were subsequently randomly selected from each township, which yielded 35 villages in total. During the last stage of the survey sampling, participants were stratified into several sex and age groups in accordance with census year 2010 in China. We enrolled, one participant from each household, ≥20 years of age, and residing at the current address for at least 5 years [20]. Among 10,759 eligible individuals, 9,404 (4,770 males and 4,634 females) completed the survey (87% response). Among respondents, 5,797 self-identified as Han (65.2%) and 3,091 as Manchu (34.78%), comprising the final analytical sample of n = 8,888.

Before the study, we obtained written informed consent from each study participant. The study protocol complied with the principles outlined in the Declaration of Helsinki. The Human Studies Committee of Sun Yat-sen University and the Liaoning province regulatory body approved the study protocol.

### Sleep quality

We used the Pittsburgh Sleep Quality Index (PSQI) to evaluate sleep quality [21]. The PSQI, a validated, self-rated, diagnostic questionnaire, consists of 19 elements, each of which is coded on a point scale of 0 to 3, and are used to create sub scores in seven component subcategories. Component variables are defined as: sleep latency on a continuous scale (time required to go from full wakefulness to sleep and the frequency of falling asleep within 30 min); habitual sleep efficiency (hours slept compared to hours spent in bed: 0, ≥85%; 1, 75–84%; 2, 65–74%; and 3, <65%); subject sleep quality (0, very good; 1, fairly good; 2, fairly bad; and 3, very bad); sleep disturbance (amount of sleep disruption and frequency of disruption); daytime dysfunction (regularity of having trouble staying awake and maintaining enthusiasm during daily activities); sleep duration (0, sleep duration >7 h; 1, 6 to ≤7 h; 2, 5 to <6 h; and 3, <5 h); and use of sleep medication (0, not used during the past month; 1, used < 1x/week; 2, 1-2x/week; and 3, ≥3x/week). A composite ‘global sleep quality score’ (0–21) is derived by adding the points for each component. A global score ≥6 distinguishes poor from good sleepers [16]. For the sub-components, the first two categories were considered as good sleep quality and the last two cases were considered as poor sleep quality. The PSQI has a diagnostic sensitivity of 89.6% and specificity of 86.5%, and the Chinese language version has a reliability coefficient of 0.82–0.83, with an acceptable test-retest reliability coefficient of 0.77–0.85 [22].

### Depression

We identified depressive symptoms using the Center for Epidemiology Scale for Depression (CES-D) [23], a depression screening tool with a validated and widely used Chinese language version [24]. Participants were asked to rate the frequency of 20 items describing depressive symptoms over the past week, on a scale of 0–3, ranging from ‘rarely or none of the time’ to ‘most or all of the time.’ A composite CES-D score (range 0–60) was derived by adding across the scores for the 20 items. A ‘positive’ screening for depressive symptoms was defined by a CES-D composite score >16 points [23]. For the CES-D survey, we excluded participants with <17 completed items to minimize bias [20].

### Covariates

A self-reported questionnaire was used to collect information regarding sociodemographic characteristics, such as age, gender, education, ethnicity, occupation, annual family income, and behavioral and lifestyle risk factors, including smoking, alcohol consumption, tea consumption, and exercise. Trained study staff recorded the weight and height of participants and calculated the body mass index (BMI; weight in kg per height in m^2^). We defined alcohol consumption as the average weekly consumption of beer, wine, and hard liquor. Current drinking was defined as alcohol consumption ≥8ml per week according to the definition from the National Institute on Alcohol Abuse and Alcoholism [24]. We defined smokers as participants who smoked at least one cigarette per day and continued for at least 1 year. We asked whether participants currently smoked (i.e., “Do you smoke currently?”). Tea consumption and habitual physical activity were defined as people who drank tea or exercised almost daily throughout the past year.

### Statistical analysis

We compared baseline characteristics between Hans and Manchus using Pearson chi-square tests for nominal variables and independent samples t-tests for continuous variables. After stratification, we also compared unadjusted baseline characteristics between good and poor sleep quality using independent samples t-tests for continuous variables, and Pearson Chi-Square tests for categorical variables. We conducted logistic regression analysis to estimate odds ratios (OR) and 95% confidence intervals (95% CI) of poor sleep quality (dependent variable) in relation to a positive depression screening (independent variable) and ethnicity in adjusted models, controlling for confounders including age, sex, smoking, exercise, family income and education [16–19]. Furthermore, we conducted logistic regression analysis to estimate the biological additive interactions between depressive symptoms and ethnicity with sleep quality [25]. The relative excess risk due to interaction (RERI), the attributable proportion due to interaction (AP), and the synergy index (S) were used to quantify the additive interactions. We also conducted the analysis stratified by ethnicity, including evaluation of augmented interaction models incorporating cross-product terms [26], to allow for heterogeneity in the effects of all covariates by race.

We conducted all statistical analyses using SPSS software (version 18.0) and SAS software (version 9.4). We considered *p*-values < 0.05 as statistically significant for a two-sided test.

## Results

### Baseline characteristics

The baseline characteristics comparing Hans and Manchus are presented in Table 1. Of 8,888 study participants, 50.95% were male and 49.05% were female, with an average age of 52.11 years, ranging from 20 to 93. Both the average (SE) CES-D score and prevalence of a positive depression screening were statistically higher (*p*<0.001) among Hans (9.03 ± 0.121 and 26.25%, respectively) than among Manchus (8.03 ± 0.162 and 22.65%, respectively). Additionally, there was a statistically significant difference (*p*<0.001) in the prevalence of poor sleep quality between the Hans (29.57%) and Manchus (20.74%). The PSQI global score and sub scores were also statistically greater among the Hans (*p*<0.001), with the exception of sleep duration. Furthermore, the Hans were more likely to smoke (*p*<0.001) and less likely to engage in habitual physical activity (*p*<0.001) than the Manchus, and had achieved a lower overall level of education (*p<*0.001).

**Table 1:** Characteristics of Han and Manchu study participants residing in rural areas of northern China, from 2012 to 2013.

	Overall (*n* = 8888)	Han (*n* = 5797)	Manchu (*n* = 3091)	*p* value^a^
Age (year)^b^	52.45 ± 13.98	53.11 ± 13.92	51.23 ± 14.02	<0.001
BMI (kg/m^2^)^b^	23.81 ± 5.14	23.86 ± 5.34	23.71 ± 4.74	0.208
PSQI score^b^	4.33 ± 2.82	4.55 ± 2.85	3.92 ± 2.71	<0.001
Subjective sleep quality	0.61 ± 0.007	0.66 ± 0.009	0.49 ± 0.011	<0.001
Sleep latency	1.24 ± 0.006	1.26 ± 0.007	1.21 ± 0.009	<0.001
Sleep duration	0.29 ± 0.007	0.30 ± 0.008	0.28 ± 0.011	0.102
Habitual sleep efficiency	0.59 ± 0.009	0.62 ± 0.011	0.54 ± 0.015	<0.001
Sleep disturbance	0.69 ± 0.006	0.72 ± 0.008	0.63 ± 0.010	<0.001
Use of sleep medication	0.15 ± 0.005	0.17 ± 0.006	0.13 ± 0.007	<0.001
Daytime dysfunction	0.75 ± 0.008	0.81 ± 0.010	0.64 ± 0.013	<0.001
Poor sleep quality^c^	2355 (26.50)	1714 (29.57)	641 (20.74)	<0.001
CES-D score^b^	8.68 ± 0.097	9.03 ± 0.121	8.03 ± 0.162	<0.001
CES-D score ≥16^c^	2222 (25.00)	1522 (26.25)	700 (22.65)	<0.001
Male sex^b^	4527 (50.95)	2959 (51.04)	1568 (50.73)	0.777
Current smoker^c^	2691 (30.27)	1833 (31.62)	858 (27.76)	<0.001
Current EtOH drinker^c^	1571 (17.68)	1055 (18.20)	516 (16.69)	0.076
Habitual tea drinker^c^	2000 (22.50)	1331 (22.96)	669 (21.64)	0.157
Habitual physical activity^c^	3487 (39.23)	2180 (37.60)	1307 (42.28)	<0.001
Annual family income^c^				0.028
<5 000 RMB	2584 (29.07)	1667 (28.76)	917 (29.67)	
5 000–10 000 RMB	3619 (40.72)	2317 (39.97)	1302 (42.12)	
10 000–30 000 RMB	2160 (24.30)	1441 (24.86)	719 (23.26)	
30 000–100 000 RMB	176 (1.98)	124 (2.14)	52 (1.68)	
>100 000 RMB	17 (0.19)	13 (0.22)	4 (0.13)	
No response	332 (3.74)	235 (4.05)	97 (3.14)	
Education^c^				<0.001
< HS	8305 (93.44)	5457 (94.13)	2848 (92.14)	
HS graduate	510 (5.74)	307 (5.30)	203 (6.57)	
> HS	73 (0.82)	33 (0.57)	40 (1.29)	
Married^c^	8503 (95.67)	5551 (95.76)	2952 (95.50)	0.576
Occupational statu^c^				0.765
Unemployed	1560 (17.55)	1021 (17.61)	539 (17.44)	
Employed	530 (5.96)	340 (5.86)	190 (6.15)	
Farmer	6559 (73.80)	4272 (73.69)	2287 (73.99)	
Retired	137 (1.54)	96 (1.66)	41 (1.33)	
Others	102 (1.15)	68 (1.17)	34 (1.10)	

Abbreviations: BMI, body mass index; CES-D, The Center for Epidemiology Scale for Depression; HS, high school; PSQI, Pittsburgh Sleep Quality Index; RMB, Chinese Yuan.

^a^ Pearson Chi-Square statistical test for nominal variables and independent samples t-test for continuous variables to test differences between Hans and Manchus.

^b^ Data presented as mean ± standard deviation.

^c^ Data presented as n (%).

### Distribution of study population characteristics across sleep quality categories in Hans and Manchus

**Fig 1:**
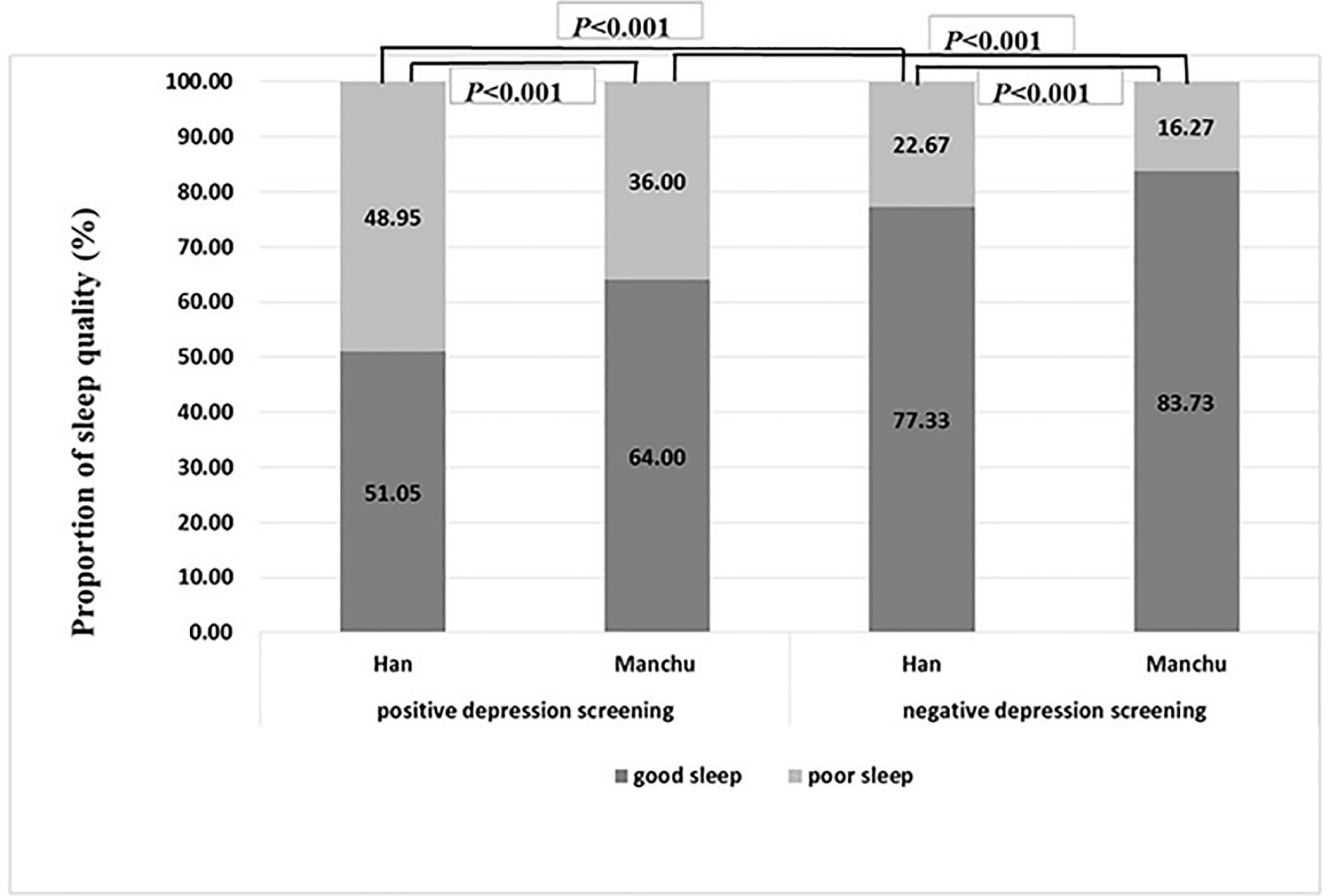
Prevalence of poor sleepers among Hans and Manchus residing in rural areas of northern China, with and without a positive depression screening, from 2012 to 2013. Chi-square statistical tests were performed to assess differences in the prevalence of poor sleep quality between groups with and without a positive depression screening, among the Hans and the Manchus.

The differences between Hans and Manchus with respect to associations between covariates and sleep quality are shown in Table 2. The CES-D scores and the prevalence of a positive depression screening were elevated among those with poor sleep quality, both in Hans and Manchus (*p*<0.001, respectively). Older age, smoking, and lower annual family income were also related to poor sleep quality in both ethnic groups. Fig 1 reveals that participants with a positive depression screening experienced a higher prevalence of poor sleep quality than those with a negative depression screening, in both ethnic groups (*p*<0.001); however, poor sleep quality overall was more prevalent in the Hans than in the Manchus (*p*<0.001).

**Table 2:** Characteristics of Han and Manchu study participants residing in rural areas of northern China, by sleep quality categories, from 2012 to 2013.

	Han			Manchu		
	Poor sleep quality (*n* = 1714)	Good sleep quality (*n* = 4083)	*p* value^a^	Poor sleep quality (*n* = 641)	Good sleep quality (*n* = 2450)	*p* value^a^
Age (year)^b^	56.98 ± 13.06	51.48 ± 13.96	<0.001	55.59 ± 12.31	50.09 ± 14.21	<0.001
BMI (kg/m^2^)^b^	23.72 ± 5.18	23.93 ± 5.40	0.191	23.71 ± 4.78	23.72 ± 4.59	0.995
CES-D score^b^	13.96 ± 0.25	6.96 ± 0.12	<0.001	13.33 ± 0.40	6.65 ± 0.16	<0.001
Depression^c^	745 (43.46)	777 (19.03)	<0.001	252 (39.31)	448 (18.28)	<0.001
Male sex^c^	845 (49.30)	869 (51.78)	0.085	305 (47.58)	1263 (51.55)	0.074
Current smoker^c^	606 (35.36)	1227 (30.05)	<0.001	194 (30.26)	664 (27.10)	<0.001
Current EtOH drinker^c^	314 (18.32)	741 (18.15)	0.877	120 (18.72)	396 (16.16)	0.122
Habitual tea drinker^c^	400 (23.33)	931 (22.80)	0.658	153 (23.87)	516 (21.06)	0.124
Habitual physical activity^c^	669 (39.03)	1511 (37.01)	0.146	282 (43.99)	1025 (41.84)	0.325
Annual family income^c^			<0.001			<0.001
<5000 RMB	597 (34.83)	1070 (26.21)		260 (40.56)	657 (26.82)	
5000–10 000 RMB	610 (35.59)	1707 (41.81)		257 (40.09)	1045 (42.64)	
10 000–30 000 RMB	397 (23.16)	1044 (25.57)		99 (15.44)	620 (25.31)	
> 30 000RMB	17 (0.99)	120 (2.93)		6 (0.94)	50 (2.05)	
N/A	93 (5.42)	142 (3.48)		19 (2.96)	78 (3.18)	
Education^c^			0.477			0.650
< HS	1605 (93.64)	3852 (94.34)		585 (91.26)	2263 (92.37)	
≥ HS	109 (6.36)	231 (5.66)		56 (8.74)	187 (7.63)	
Married^c^	1629 (95.04)	3922 (96.06)	0.08		2334 (95.26)	0.212
Occupational status^c^			0.878			0.408
Farmer	1263 (73.69)	3009 (73.70)		475 (74.10)	1812 (73.96)	
other	451 (26.31)	1074 (26.30)		166 (25.90)	638 (26.04)	

Abbreviations: BMI, body mass index; CES-D, The Center for Epidemiology Scale for Depression; HS, high school; RMB, Chinese Yuan.

^a^ Pearson Chi-Square statistical test for nominal variables and independent samples t-test for continuous variables to assess differences between groups with and without sleep disorder.

^b^ Data presented as mean ± standard deviation.

^c^ Data presented as n (%).

### Association of sleep quality with positive depression screening and ethnicity

In Tables 3 and 4, poor sleep quality, including global sleep quality and sub-elements of the PSQI, were determined as outcomes. As shown in Table 3, adjusted for ethnicity and other covariates, positive depression symptoms were associated with higher odds of poor sleep quality, from 153% to 623% compared to no depression symptoms, especially of sleep disturbance (OR = 5.15; *95% CI*, 4.27–6.22) and use of sleep medication (OR = 6.23; *95% CI*, 4.54–8.54). According to ethnicity, adjusted for depressive symptoms and other covariates, the ORs of global poor sleep quality was 34% lower in Manchus than in Hans (OR = 0.66; *95% CI*, 0.59–0.74). The results were similar for the sub-elements of the PSQI except for sleep duration.

**Table 3:** Adjusted odds ratios (95%CI) of poor sleep quality in relation to positive depression screening and ethnicity among Han and Manchu study participants residing in rural areas of northern China, m 2012 to 2013.

	Global sleep quality	Subjective sleep quality	Sleep latency	Sleep duration	Habitual sleep efficiency	Sleep disturbance	Use of sleep medication	Daytime dysfunction
	OR(95% CI)	OR(95% CI)	OR(95% CI)	OR(95% CI)	OR(95% CI)	OR(95% CI)	OR(95% CI)	OR(95% CI)
Depressive^a^ symptoms								
without (reference)	1.00	1.00	1.00	1.00	1.00	1.00	1.00	1.00
with	2.90(2.61–3.22)	3.39(2.88–3.99)	2.62(2.35–2.93)	1.53(1.27–1.85)	1.29(1.12–1.47)	5.15(4.27–6.22)	6.23(4.54–8.54)	3.01(2.66–3.42)
Ethnicity^b^								
Han(reference)	1.00	1.00	1.00	1.00	1.00	1.00	1.00	
Manchus	0.66(0.59–0.74)	0.73(0.59–0.90)	0.78(0.69–0.89)	0.97(0.80–1.17)	0.88(0.77–1.00)	0.72(0.58–0.89)	0.66(0.47–0.94)	0.75(0.65–0.86)

^a^ Odds ratios (ORs) adjusted for age, sex, smoking, annual family income, and ethnicity

^b^ Odds ratios (ORs) adjusted for age, sex, smoking, annual family income, and depressive symptoms.

**Table 4:** Adjusted ORs (95%CI) of poor sleep quality in relation to the joint effects of positive depression screening and ethnicity among Han and Manchu study participants residing in rural areas of northern China, from 2012 to 2013 ^a^.

	Global sleep quality	Subjective sleep quality	Sleep latency	Sleep duration	Habitual sleep efficiency	Sleep disturbance	Use of sleep medication	Daytime dysfunction
	OR(95% CI)	OR(95% CI)	OR(95% CI)	OR(95% CI)	OR(95% CI)	OR(95% CI)	OR(95% CI)	OR(95% CI)
Manchu without depression (REF)	1.00	1.00	1.00	1.00	1.00	1.00	1.00	1.00
Manchu with depression	2.61(2.16–3.17)	2.86(2.10–3.89)	2.75(2.26–3.35)	2.24(1.63–3.08)	1.36(1.07–1.73)	3.80(2.67–5.41)	4.46(2.44–8.12)	2.69(2.13–3.41)
Han without depression	1.44(1.26–1.64)	1.21(0.99–1.55)	1.27(1.10–1.46)	1.28(1.01–1.63)	1.19(1.02–1.39)	1.10(0.81–1.45)	1.15(0.66–1.99)	1.27(1.07–1.52)
Han with depression	4.37(3.76–5.08)	4.38(3.43–5.60)	3.25(2.78–3.80)	1.63(1.24–2.16)	1.49(1.24–1.80)	6.39(4.79–8.52)	8.09(4.92–13.32)	4.07(3.34–4.83)

^a^ Odds ratios (OR) adjusted for age, sex, smoking, and family income.

### Biological additive interaction between depressive symptoms and ethnicity with sleep quality

As shown in Tables 4 and S1, we estimated the individual and joint effects of depressive symptoms and ethnicity. We identified additive interactions between depressive symptoms and ethnicity with global sleep quality and four sub-PSQI elements, including subjective sleep quality, sleep disturbance, use of sleep medication, and daytime dysfunction. For example, we found a 132% (95% CI, 70%-194%) excess odds for global poor sleep quality among Hans with depressive symptoms compared to sum of the individual effects of Han ethnicity and depressive symptoms, and the attributable proportion due to interaction (AP) of global poor sleep quality among Hans with depressive symptoms was 30.2% (95% CI, 17.9%-42.5%).

## Discussion

### Key findings of the present study

In this population-based study of close to 9,000 participants in northeastern rural China, we found high levels of poor sleep quality and a high proportion of positive depression screenings, among both Chinese Hans and Manchus. Our findings revealed that the prevalence of poor sleep quality and depression was lower among the Manchus than among the Hans. Depression was associated with higher odds of poor sleep quality.

Findings from our study, using a large and representative sample of rural participants, are important for multiple reasons. First, the prevalence of positive depression screenings and poor sleep quality among the Manchu ethnic minority in northeastern China’s Liaoning province, was, to the best of our knowledge, determined for the first time, and was lower than for the Han ethnic majority. Second, this is one of few studies to date, to obtain self-reported sleep quality information from adults residing in rural China, and the only study to use these data to demonstrate associations between depressive symptoms and poor sleep quality in the Han and Manchu populations. Of note, this association was conditionally independent of known sociodemographic and behavioral risk factors for sleep disorders. Third, we employed a comprehensive assessment tool, the PSQI scale, to capture the multidimensional aspect of sleep quality more effectively, compared to prior publications that focused on only sleep duration or sleep disturbance [27–29]. Overall, the present study revealed concerns about sleep disorders and mental health problems in vulnerable rural Chinese populations, which has scarcely been studied before.

### Depression prevalence and sleep quality in rural China

For over 50 years now, systematic epidemiological studies revealed a growing prevalence of depression worldwide [30]. However, the majority of these studies have focused on developed countries and on urban areas [7, 31]. Zhou et al. reported that, in rural areas in China, the prevalence of depressive symptoms among the residents aged ≥35 years was 5.9%, greater than for cities in southern China [32]. In contrast, our present study found a prevalence of 22.65% and 26.25% for positive depression screenings, in the Manchus and the Hans, respectively. Although our use of a highly sensitive depression screening tool, rather than a highly specific diagnostic tool, may have somewhat inflated estimates, our data with a large, representative population sample suggested that depression may be a more serious health problem in the rural Chinese populations than previously thought. A recent study of rural adults in Deqing, China reported that the prevalence of poor sleep quality was 27.7% (95% CI = 25.4–29.7%), which was similar to our study s [33].

The differences among these studies could be related to various factors, including use of different assessment scales, and different sizes and the sociodemographic profiles of the study populations. Thus, studies with larger populations and optimized assessments of depression and sleep quality are needed to clarify the relation between depression and sleep in rural Chinese populations.

### An interaction between depression and ethnicity

The prevalence of poor sleep quality among the Manchu ethnic minority was reported for the first time in the present study. We also detected differences in the prevalence of poor sleep quality for Han and Manchu ethnicities. The results of the interaction analysis showed excess odds for global poor sleep quality among Hans with depressive symptoms compared to the sum of the individual effects of Han ethnicity and depressive symptoms, which indicated an additive interaction between depressive symptoms and ethnicity with global sleep quality. We found similar additive interactions for four sub-PSQI elements, including subjective sleep quality, sleep disturbance, use of sleep medication, and daytime dysfunction. Manchus have a different cultural background from and tend to have different health-related behaviors than Hans, such as more habitual physical activity and fewer smokers as we found in the present study. We also found an interaction between ethnicity and habitual physical activity, in which the protective association against a positive depression screen was stronger in Manchus than in Hans (OR = 0.83; 95%*CI*, 0.77–0.89). Thus, a higher level of physical activity may contribute to lower prevalence of positive depression screening in Manchus and also the lower prevalence of poor sleep quality in Manchus.

Our findings were mostly consistent with those from previous studies, which indicated that demographic, family, lifestyle, and social factors affected sleep disorders and depressive symptoms [16–19]. However, risk factors for sleep disorders and depression varied across these prior studies. For example, the cross-sectional Multi-Ethnic Study of Atherosclerosis showed that associations between sleep disturbance and depression could be modified by education and sex [16]. Another cross-sectional study of 258,793 adults reported a significantly greater odds of depression (OR = 1.54; 95%*CI*, 1.37–1.73) and sleep problems (OR = 1.37; 95%*CI*, 1.25–1.50) among caregivers, and that these associations tended to be stronger in high-income countries [18]. Thus, lifestyle and social changes may be useful for designing interventions to reduce depression and sleep disorders in rural China. For more definitive data, a large, prospective investigation of depression and sleep quality should be performed in the future, that captures a wider array of social and lifestyle factors and includes more ethnic minorities than considered here.

### The association between sleep and depression

U.S. studies have shown that sleep disturbances may be an early indicator for depression, which can likewise be a sign of sleep disturbance [16, 34–36]. A study of depression in the Netherlands (NESDO) found sleep disturbance in 59.9% of depressed older persons [19]. A new China Kadoorie Biobank (CKB) study, including 512,891 adults aged 30–79 years, showed that sleep disturbance increased the odds of depression by 3.31 to 4.17 times [37]. Other studies from Asia have corroborated this finding as well. For instance, a population-based 4-year cohort study from Taiwan confirmed that relapse insomnia increased the risk of anxiety and depression [38]. Another recent study of a young and healthy nurse population in south China also identified depression as an independent risk factor for excessive daytime sleepiness, with an adjusted odds ratio of 2.24 (95%*CI* 1.51–3.31) [28]. The results of the current study were consistent with these previous studies, in demonstrating a close association between depression and poor sleep, but not indicating a cause-effect relationship.

The biological mechanisms that contribute to the widely reported comorbidity between poor sleep and depression are uncertain. Chen et al. reported that sleep/circadian timing is dependent upon several neurotransmitter systems, including norepinephrine (NE) and 5-hydroxytryptophan (5-HT), which are also critical to mental, emotional, and cognitive function [39]. More recently, a study in adult male rats found that sleep loss has prolonged effects on the activity of multiple hypothalamic areas, indicating that hypothalamic changes underlie the well-established relationship between sleep loss and several diseases, possibly including depression [40]. Further research on neurobiological assessments, homeostatic dysregulation, and emotional and physical hyperarousal should help to shed light on the biologic mechanisms responsible for epidemiologic associations between poor sleep quality and depressive symptoms.

### Strengths and limitations

There were several strengths in the current study. We evaluated a relatively large randomly selected sample of rural participants from ethnically diverse backgrounds, using systematic and validated measures of sleep quality and depressive symptoms, and incorporated adjustment for confounding by important demographic and lifestyle factors. However, several limitations to our findings should be noted as well. The use of self-reported data might be susceptible to a reporting bias, leading to misclassification for some participants. Unfortunately, other methods to objectively assess sleep, such as polysomnography, are prohibitively costly for use with large population based studies. Still, the PSQI is a validated and widely used tool for population-based investigations [21]. Similarly, the CES-D is a screening tool, rather than a diagnostic tool, and so is likely to have falsely classified some participants as positive [23]. Thus, our results should not be interpreted in the context of clinical depression, and so a future investigation incorporating clinical diagnosis will be necessarily to corroborate these findings. Additionally, using a cross-sectional study design did not allow us to establish temporality, in which we cannot be sure if the onset of depressive symptoms preceded poor sleep, or vice versa. A prospective investigation will be necessary to more clearly evaluate sequence of this association.

## Conclusions

Poor sleep quality and depression may be major, yet underappreciated, public health problems in northeastern China’s rural populations. Ethnicity may also be related to the prevalence of both problems, via differences in health-related behaviors between Hans and Manchus, or possibly associated with presently unrecognized biological or social factors. Depressive symptoms were associated with a higher odds of poor sleep quality in both groups, with some variation in the associations among the Hans and the Manchus. A prospective population-based cohort investigation is needed to validate our findings and to rule out additional confounding factors. Future studies to explore the reasons behind the higher prevalence of depressive symptoms and poor sleep quality among rural Chinese compared to urban Chinese are also necessary. Still, our study provides baseline data not only for use in designing future investigations, but also for mental health professionals to use in designing interventions to reduce the prevalence of depressive symptoms and poor sleep quality in this rural Chinese population.

## Supporting information

S1 Table Measures of additive interaction of positive depression screening and ethnicity on poor sleep quality among Han and Manchu study participants residing in rural areas of northern China, from 2012 to 2013. (DOCX)

S1 File Study questionnaire. (DOCX)
