# Supplementary material for: Association between depressive symptoms and poor sleep quality among Han and Manchu ethnicities in a large, rural, Chinese population
Source: PLoS One. 2019 Dec 19;14(12):e0226562. doi: 10.1371/journal.pone.0226562 (PMC6922383; doi:10.1371/journal.pone.0226562)
Supplement: S1 Table — (DOCX) [file pone.0226562.s001.docx]

Association between depressive symptoms and poor sleep quality among Han and Manchu ethnicities in a large, rural, Chinese population

Ru-Qing Liu^1^, Michael S. Bloom^1,2, 3^, Qi-Zhen Wu^1^, Zhi-Zhou He^1^, Zhengmin Qian^4^, Katherine A. Stamatakis^4^, Echu Liu^5^, Michael Vaughn^6^, Wayne R. Lawrence^3^, Mingan Yang^7^, Tao Lu^8^, Qian-Sheng Hu^1*^, Guang-Hui Dong^1*^

**S1 Table.**

**Measures of additive interaction of positive depression screening and ethnicity on poor sleep quality among Han and Manchu study participants residing in rural areas of northern China, from 2012 to 2013.**

|  | Global sleep quality | Subjective sleep quality | Sleep latency | Sleep duration | Habitual sleep efficiency | Sleep disturbance | Use of sleep medication | Daytime dysfunction |
| --- | --- | --- | --- | --- | --- | --- | --- | --- |
|  |  |  |  |  |  |  |  |  |
|  | E(L, U) | E(L, U) | E(L, U) | E(L, U) | E(L, U) | E(L, U) | E(L, U) | E(L, U) |
| RERI | 1.32(0.70, 1.94) | 1.35(0.45, 2.24) | 0.25(-0.32, 0.82) | -0.90(-1.65, -0.14) | -0.06(-0.44, 0.32) | 2.54(1.19, 3.89) | 3.56 (0.85, 6.27) | 1.09(0.41, 1.77) |
| AP | 0.30(0.18, 0.42) | 0.31(0.12, 0.49) | 0.08(-0.10, 0.25) | -0.55(-1.03, -0.08) | -0.04(-0.30, 0.22) | 0.40(0.22, 0.57) | 0.44 (0.18, 0.69) | 0.27(0.12, 0.42) |
| S | 1.64(1.27, 2.12) | 1.65(1.11, 2.45) | 1.12(0.85, 1.48) | 0.41(0.22-0.77) | 0.89(0.44, 1.80) | 1.88(1.27, 2.78) | 1.98 (1.11, 3.54) | 1.56(1.14, 2.15) |

Abbreviations: RERI, the relative excess risk due to interaction; AP, the attributable proportion due to interaction; S, the synergy index; E(L, U), estimate (lower, upper).
