# Supplementary material for: Association between depressive symptoms and poor sleep quality among Han and Manchu ethnicities in a large, rural, Chinese population
Source: PLoS One. 2019 Dec 19;14(12):e0226562. doi: 10.1371/journal.pone.0226562 (PMC6922383; doi:10.1371/journal.pone.0226562)
Supplement: S1 File — (DOCX) [file pone.0226562.s002.docx]

Association between depressive symptoms and poor sleep quality among Han and Manchu ethnicities in a large, rural, Chinese population

Ru-Qing Liu^1^, Michael S. Bloom^1,2, 3^, Qi-Zhen Wu^1^, Zhi-Zhou He^1^, Zhengmin Qian^4^, Katherine A. Stamatakis^4^, Echu Liu^5^, Michael Vaughn^6^, Wayne R. Lawrence^3^, Mingan Yang^7^, Tao Lu^8^, Qian-Sheng Hu^1*^, Guang-Hui Dong^1*^

**S2 File. Study questionnaire.**

**调查表编号：**□ □ □ □ □ □ □ □

**辽宁省城市居民睡眠状况和高血压调查表**

姓名name 性别sex 年龄age

民族ethnicity 婚否marriage status

如为女性请填写：生育子女数 ； 是否绝经：否=1 是=2； 绝经年龄： 岁

**体格检查**

- - 1. 身高height（cm） 体重weight（kg）
    2. 腰围waist circumference（cm） 臀围hip circumference（cm）
    3. 心率heart rate： （次/分钟times per minute）

血压：收缩压(mmHg) 舒张压(mmHg)

您是否正在服用降压药？ 否=1 是=2

诊断：高血压：否=1 是=2

**一、一般情况demographic characteristics**

1. 文化程度education：文盲illiteracy =1 小学primary school=2 初中Junior high school =3

高中或中专high schoo =4 大专或大专以上college or above=5

1. 劳动强度labor intensity：极轻度very mild=1 轻度mild=2 中度moderate=3 重度severe=4
2. 职业occupation：干部cadres=1 工人worker=2 农民farmer=3 知识分子the-intellectual =4

家庭妇女house wife=5 退休retirement=6 无业jobless=7 其它others=8

1. 家庭年收入annual family income：5000元以下less than ¥5 000=1 5千-1万 ¥5 000-10 000 =2

1-3万 ¥10 000-30 000=3 3-10万¥30 000-100 000=4

10万以上more than ¥100 000=5 不知道unknown=9

1. 对目前经济状况的满意程度：不满意=1 基本满意=2 比较满意=3 很满意=4
2. 家庭人口数：＿＿ 人

**二、高血压相关情况**

1. 您认为高血压对生命构成严重威胁吗？无所谓=1 有一些=2 非常重要=3
2. 高血压家族史：父亲=1 母亲=2 兄弟姐妹=3 没有=4
3. 您以前是否患有高血压？否=1 是=2 如“是”，请回答以下问题：
4. 当时诊断血压水平：收缩压(mmHg) 舒张压(mmHg)
5. 患有高血压后是否定期测量血压：没再测量过=1 每周至少1次=2 至少每月1次=3

至少3个月1次=4 半年1次=5 半年以上1次=6 记不清=7

1. 因高血压就诊的次数（包括初诊在内） 次/年
2. 未就诊原因（可多选）：认识不足=1 经济困难=2 工作忙没时间=3 交通不便怕麻烦=4 其它=5
3. 坚持服药情况：规律(>9个月/年)=1 间断(3~9个月/年)=2 偶尔(<3个月/年)=3 未服药=4
4. 服用降压药的降压效果：

优良(全年3/4以上时间血压140/90mmHg以下)=1 尚可(全年1/2以上时间血压140/90mmHg以下)=2

不良(全年1/2以下上时间血压超过140/90mmHg)=3 未测量过血压=4

1. 具体降压药费用 元/年 每年高血压治疗费用 元/年
2. 未服用降压药的原因：认识不足=1 经济困难=2 缺乏指导=3 太麻烦或经常忘记=4 其它=5

**三、吸烟饮酒情况smoke and drink**

1. 您是否吸烟？否=1 是=2 吸烟量 支/天 烟龄 年

Do you smoke currently? No=1 Yes=2 cigarette consumption pieces per day

How long have you been smoking? years

1. 您是否戒烟？否=1 是=2 戒烟的累积时间：<6个月=1 6~12个月=2 >1年=3 ≥3年=4
2. 您是否嗜酒？否=1 是=2 您开始饮酒的年龄 岁

Do you drink currently? No=1 Yes=2 the age began to drink years old

| 饮酒种类items | 饮酒量/次  alcohol consumption each time | 次/周  times per week | 周/月  weeks per month | 备注note |
| --- | --- | --- | --- | --- |
| 白酒 hard liquor | ml/次each time |  |  |  |
| 啤酒 beer | ml/次each time |  |  |  |
| 红酒及其他 wine | ml/次each time |  |  |  |

1. 您是否戒酒？否=1 是=2 戒酒的累积时间：<6个月=1 6~12个月=2 >1年=3 ≥3年=4

**四、体育锻炼和饮食 exercises and diet**

1. 您每天参加运动项目吗？否=1 是=2

Do you do physical activities daily? No=1 Yes=2

1. 运动项目是：①散步 ②慢跑 ③气功或太极拳 ④健身操(舞) ⑤球类 ⑥游泳 ⑦其它

Sports: ① take a walk ② jogging ③ Tai Chi ④ aerobics ⑤ balls ⑥swimming ⑦ others

1. 您每次锻炼平均花费时间 小时 您每天看电视的时间 小时

The average time for each exercise hours the average time for TV hours per day

1. 您是否有意控制饮食(低油,低热)：①基本不会 ②偶尔会 ③经常会 ④每天注意
2. 饮用含糖饮料的频率： ①一周有5天以上 ②一周有2~4天 ③一周少于等于1天
3. 请选择主食的种类：①米饭 ②面食 ③面条 ④烙饼 ⑤其它
4. 每周吃新鲜蔬菜量：①很少吃 ②2两以下 ③半斤-2斤 ④2斤以上
5. 每周吃瘦肉、鱼、禽类量：①很少吃 ②半斤以下 ③半斤-1斤 ④1斤以上
6. 每周吃油腻食物天数(肥肉、油炸食品)：①<1天 ②1-2天 ③3-4天 ④5-7天
7. 每周吃咸菜或大酱：①很少吃 ②每天1次 ③每天2次 ④每天3次及以上
8. 每月购买或使用食盐量： 斤/月
9. 您是否喝茶：否=1 是=2 饮茶量 两/年

Do you drink tea daily? No=1 Yes=2 tea consumption each time liang per year

1. 您是否吃早餐？①几乎不吃 ②每周吃1-2天 ③每周吃3-5天 ④几乎每天都吃

**五、病史**

1. 冠心病：否=1 心绞痛=2 心肌梗塞=3 心律失常=4 其它或不详=5
2. 脑卒中：否=1 脑出血=2 脑梗塞(含脑血栓和脑栓塞)=3 蛛网膜下腔出血=4 其它或不详=5
3. 糖尿病：否=1 I型糖尿病=2 II型糖尿病=3 其它或不详=4
4. 肾病： 否=1 是=2

六、**睡眠情况调查（最近1个月情况）**

1. 您通常每晚能睡几小时？①大于9小时 ②7-8小时 ③5-6小时 ④不足4小时
2. 您有失眠吗？①没有 ②偶尔失眠 ③失眠
3. 家庭其他成员有失眠情况吗？①无 ②父亲失眠 ③母亲失眠 ④兄弟姐妹失眠 ⑤其他亲戚失眠
4. 您睡眠时有无呼吸困难或呼吸暂停现象？①没有 ②偶尔有 ③经常有 ④不知道
5. 您平时睡下后多少时间睡着？①一沾枕头就睡着 ②30分钟左右睡着 ③1小时后才睡着
6. 睡眠时夜间易醒或早醒 ①无 ② <1次/周 ③ 1~2次/周 ④ ≧3次/周
7. 您睡眠时是否打呼噜？是=1 否=2

Note: the following is the Chinese version of the Pittsburgh Sleep Quality Index (PSQI)

下面一些问题是关于您最近**一个月的睡眠状况**，请填写或选择最符合您实际情况的答案。

1、 近1个月，晚上上床睡觉时间通常是 点钟。

2、 近1个月，从上床到入睡通常需要 分钟。

3、 近1个月，通常早上 点起床。

4、 近1个月，每夜通常实际睡眠时间 小时

对下列问题请选择一个最适合您的答案。

5、 近一个月，您有没有因下列情况影响睡眠而烦恼

a. 入睡困难（30分钟内不能入睡） ①无 ② <1次周 ③ 1~2次周 ④ ≧3次周

b. 夜间易醒或早醒 ①无 ② <1次/周 ③ 1~2次/周 ④ ≧3次/周

c. 夜间去厕所 ①无 ② <1次/周 ③ 1~2次/周 ④ ≧3次/周

d. 呼吸不畅 ①无 ② <1次周 ③ 1~2次周 ④ ≧3次周

e. 咳嗽或鼾声高 ①无 ② <1次周 ③ 1~2次周 ④ ≧3次周

f. 感觉冷 ①无 ② <1次周 ③ 1~2次周 ④ ≧3次周

g. 感觉热 ①无 ② <1次周 ③ 1~2次周 ④ ≧3次周

h. 做噩梦 ①无 ② <1次周 ③ 1~2次周 ④ ≧3次周

i. 疼痛不适 ①无 ② <1次周 ③ 1~2次周 ④ ≧3次周

j. 其他影响睡眠的事情 ①无 ② <1次周 ③ 1~2次周 ④ ≧3次周

如果有，请说明：

6、 近1个月，总的来说，您认为自己的睡眠质量 ①很好 ②较好 ③较差 ④很差

7、 近1个月，您用催眠药物的情况 ①无 ② <1次周 ③ 1~2次周 ④ ≧3次周

8、 近1个月，您感到困倦吗? ①无 ② <1次周 ③ 1~2次周 ④ ≧3次周

9、 近1个月，您感到做事的精力不足吗①很好 ② 较好 ③ 较差 ④ 很差

Note: the following is the Chinese version of the Center for Epidemiological Survey, Depression Scale (CES-D)

想想最近一星期你有几天有下面所描述的感觉。请在每一问题后填写上适合你的等级。

**A． 0天**

**B． 1天**

**C． 2天**

**D． 3天**

**E． 4天**

**F． 5天**

**G． 6天**

**H． 7天**

1. 我经受着平常没有的烦扰。
2. 我不想吃东西：我食欲很差。
3. 即使在家人的帮助下，我也感到无法甩掉忧郁的心境。
4. 我觉得自己象大家一样好。
5. 我不能集中精力做事情。
6. 我感到压抑。
7. 我觉得自己做什么都很费劲。
8. 我对未来充满希望。
9. 我觉得自己的生活很失败。
10. 我感到害怕。
11. 我失眠。
12. 我比平常说话少。
13. 我感到孤独。
14. 人们不友好。
15. 我感到生活是享受。
16. 我有时哭泣。
17. 我感到悲伤。
18. 我觉得大家不喜欢我。
19. 我无法提起精神。
20. 我很快乐。

以下是关于人的一些状态的描述，每个问题都有4个可选择的答案：

**A.几乎没有 B.有些 C.经常 D.几乎总是如此**

请您按你的情况，选择一个最符合你的答案，并填写在题号前的横线上。

1、我感到愉快。

2、我感到神经过敏和不安。

3、我感到自我满足。

4、我希望能象别人那样地高兴。

5、我感到我象衰竭一样。

6、我感到很宁静。

7、我是平静的、冷静的和泰然自若的。

8、我感到困难一一堆集起来，因此无法克服。

9、我过分忧虑一些事，实际这些事无关紧要。

10、我是高兴的。

11、我的思想处于混乱状态。

12、我缺乏自信。

13、我感到安全。

14、我容易做出判断。

15、我感到不适合。

16、我是满足的。

17、一些不重要的思想总缠绕着我，并打扰我。

18、我产生的沮丧是如此强烈，以至我不能从思想中排除它们。

19、我是一个镇定的人。

20、当考虑我目前的事情和利益时，我就陷入紧张状态。

**调查到此结束，请您从头检查一下是否有遗漏和错误。非常感谢您认真帮助我们完成这次调查。**
